# Bioactive peptides identification and nutritional status ameliorating properties on malnourished rats of combined eel and soy-based tempe flour

**DOI:** 10.3389/fnut.2022.963065

**Published:** 2022-09-28

**Authors:** Nindy Sabrina, Mochammad Rizal, Fahrul Nurkolis, Hardinsyah Hardinsyah, Melvin Junior Tanner, William Ben Gunawan, Matthew Nathaniel Handoko, Nelly Mayulu, Nurpudji Astuti Taslim, Dwi Sari Puspaningtyas, Sutamara Lasurdi Noor, Vincentius Mario Yusuf, Happy Kurnia Permatasari, Son Radu

**Affiliations:** ^1^Department of Nutrition, Dietetics, and Food, Faculty of Medicine, Nursing, and Health Sciences, Monash University, Melbourne, VIC, Australia; ^2^Nutrition Program, Faculty of Food Technology and Health, Sahid University of Jakarta, South Jakarta, Indonesia; ^3^Division of Nutritional Sciences, Cornell University, Ithaca, NY, United States; ^4^Department of Biological Sciences, Faculty of Sciences and Technology, State Islamic University of Sunan Kalijaga (UIN Sunan Kalijaga Yogyakarta), Yogyakarta, Indonesia; ^5^Applied Nutrition, Faculty of Human Ecology, IPB University, Bogor, Indonesia; ^6^Department of Nutrition, Faculty of Public Health, University of Indonesia, Depok, Indonesia; ^7^Department of Nutrition Science, Faculty of Medicine, Diponegoro University, Semarang, Indonesia; ^8^Nutrition and Food, Faculty of Medicine, Sam Ratulangi University, Manado, Indonesia; ^9^Clinical Nutrition, Faculty of Medicine, Hasanuddin University, Makassar, Indonesia; ^10^Human Nutrition, Bloomberg School of Public Health, The Johns Hopkins University, Baltimore, MD, United States; ^11^Clinical and Public Health Nutrition Programme, University College London, London, United Kingdom; ^12^Department of Biochemistry and Biomolecular, Faculty of Medicine, Brawijaya University, Malang, Indonesia; ^13^Department of Food Sciences, Universiti Putra Malaysia, Selangor Darul Ehsan, Malaysia

**Keywords:** eel, tempe, serum protein, hemoglobin, IGF-1, undernutrition, malnutrition

## Abstract

**Background and aims:**

A combined eel and soy-based tempe (CEST) flour is rich in nutrients, especially its high amino acid content in which bioactive peptides (BPs) are expected to be found. Hence, this research aimed to identify the BPs of CEST flour and CEST supplementation’s effect on improving nutritional status biomarkers by ameliorating serum protein, hemoglobin, and IGF-1 of malnourished rats.

**Methods:**

CEST flour with a ratio of eel and soy-based tempe of 1:3.5 was produced by applying the oven drying method. Amino acid sequences from six BPs were analyzed using a protein sequencer and spectrometer-electrospray ionization (MS-ESI). A total of thirty malnourished male *Rattus norvegicus* aged 3–4 weeks were given low-protein (LP; 4% w/w protein) diet treatment for 4 weeks. Afterward, rats were divided into 3 groups of 10 rats. Group A and B remained on a low-protein diet for 4 weeks, receiving an LP diet and getting doses of CEST of 100 and 200 mg/kg BW, respectively, *via* oral. Group C or control was given a Normal-protein (NP) diet (23% w/w of protein) and was allowed to feed *ad libitum* during the trial period without a dose of CEST.

**Results:**

Six bioactive peptides were found, with WMGPY being the most abundant, along with a DPPH radical scavenging activity of 5.0 mg/mL. The results showed that serum protein, hemoglobin, and IGF-1 of group B were significantly higher compared to groups A and C (*p* = 0.0021). CEST dose of 200 mg/kg BW was more effective to increase serum levels of protein (*p* = 0.0052), hemoglobin, and IGF-1 (*p* < 0.0001) compared to a 100 mg/kg BW dose.

**Conclusion:**

This indicates that the CEST flour has six bioactive peptides, which may contribute to the improvement of nutritional status biomarkers. To establish its potential impact, a human clinical study is urgently needed.

## Graphical Abstract

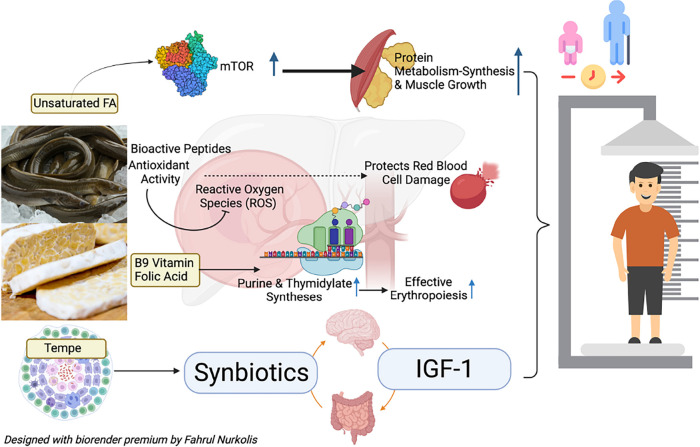

## Introduction

The Asian swamp eel (*Monopterus albus*) and tempe are food sources of protein. Adding to the value, the Asian swamp eel is rich in various minerals (1, 2). On the other hand, tempe has the highest protein content than other plant-based protein sources (2). Both products have been processed into flour and combined with a specific formulation resulting in high protein content (57.08 ± 0.08%) with high essential and non-essential amino acids, vitamin B9, and unsaturated fatty acid content based on preliminary research (3). Those mentioned properties possess the potential to alleviate malnutrition, especially its high amino acid content which is rich in bioactive peptides with various health benefits. Bioactive peptides are fragments of protein (4). Hence, the higher the protein and amino acid content, the higher the chances of containing bioactive peptides.

Stunted children possess lower serum amino acids than non-stunted children (5). Amino acid supplementation, especially aromatic amino acids, accelerates net protein synthesis in children with severe acute malnutrition during catch-up growth treatment (6). Polyunsaturated fatty acids (PUFAs) are required for tissue growth and immune function. PUFA is lower in severely acutely malnourished children than in non-malnourished children (7). Vitamin B9 (folate) deficiency could cause megaloblastic anemia and elevated homocysteine levels that cause cardiovascular diseases (8, 9). Hence, folate deficiency could worsen the outcomes of malnutrition. The results mentioned above from the preliminary research and supporting findings from other studies open a new opportunity to further test combined eel and soy-based tempe (CEST’s) potential to alleviate malnutrition.

Malnutrition (undernutrition) causes certain biomarkers levels to drop. For example, undernutrition causes a decrease in serum protein, hemoglobin, and insulin-like growth factor-1 (IGF-1). Serum protein decrease is caused by inadequate intake of dietary protein, it was proven that malnourished patients possess a lower serum protein level than non-malnourished patients (10). Malnutrition, especially stunting, is strongly associated with low hemoglobin levels (anemia). Malnutrition causes the host to have a weaker immune system, thus resulting in vulnerability to infection and inflammation that lowers hemoglobin levels (11). IGF-1 is a marker for undernutrition and can be studied in a malnutrition context. During chronic undernutrition, growth hormone signaling and secretion are reduced, thus reducing IGF-1 levels (12). IGF-1 limits cholesterol accumulation *via* the activation of insulin receptors and IGF-1 receptors themselves (13, 14). IGF-1 also modulates lipid production, one of which is through the Sterol Response Element-Binding Protein-1 mechanism (15). Furthermore, the increase in IGF-1 can also be modulated by the diversity of the gut microbiome, which can occur with the consumption of synbiotics (a combination of probiotics and prebiotics) (16), such as soy-based tempe. Childhood stunting and wasting may pose a risk of cardiovascular diseases, such as cholesterol accumulation (17).

Alleviating malnutrition could be achieved by increasing the aforementioned biomarkers to the normal value. Combined eel (*M. albus*) and soy-based tempe (CEST) flour supplementation is a potential strategy for alleviating malnutrition. Based on preliminary research, CEST is rich in protein, amino acids, unsaturated fatty acids, and vitamin B9 contents. The *in vivo* experiment was conducted using malnourished rats as a subject of intervention. Hence, this research aims to identify bioactive peptides of a combined eel (*M. albus*) and soy-based tempe (CEST) flour and the CEST supplementation effect on improving nutritional status biomarkers by ameliorating serum protein, hemoglobin, and IGF-1 of malnourished rats.

## Materials and methods

### Asian swamp eel sample preparation

One kilogram of Asian swamp eel (*M. albus*) was purchased from a local market in Jakarta. The sample was cleaned, steamed for 10 min, and the bones were removed. The material was dried in a 60°C oven for 12 h before being pulverized for analysis. A 60-mesh filter was used to filter the dried sample. The formulation and overall research were carried out at the Laboratory of the Ministry of Health Polytechnic Jakarta II (Poltekkes Kemenkes Jakarta II), Jakarta 12,540, Indonesia.

### Tempe sample preparation

One kilogram of soybean [*Glycine max (L.)* Merr.] tempe was purchased from a local market in Jakarta. The entire sample was then cut into a narrow square and cooked for 20 min. After steaming, the sample was baked for 12 h at 60°C before pulverizing. A 60-mesh filter was used to filter the sample.

### Formulation of combined eel and soy-based tempe flour

Swamp eel and tempe flour samples were combined in a ratio of 1:3.5 since this formulation has the highest vitamin B9 or folic acid and unsaturated fatty acid content according to prior research (3). Homogenization was done using a Sinmag planetary mixer.

### Analysis of sequences and molecular weights of amino acids

A protein sequencer (Applied Biosystems 494) was used to examine the amino acid sequences of six bioactive peptides (BPs) (BP1 to BP6) (Applied Biosystems Inc., Foster City, CA, USA). A time-of-flight quadrupole mass spectrometer (MS) paired with an electrospray ionization (ESI) source determines the molecular weight of the six BPs (BP1 to BP6). The analysis of sequences and molecular weights of amino acids or BPs was performed according to the method described by Zhang et al. (18). One of the most abundant BPs in CEST flour was identified, and inhibitory activity against DPPH was assessed (18).

### Antioxidant activity (2,2-diphenyl-1-picrylhydrazyl radical scavenging activity)

According to Kaur et al. (19) and Permatasari et al. (20), the percent (%) inhibition of 2,2-diphenyl-1-picrylhydrazyl (DPPH) reagent by CEST flour was performed. In the testing vial (at a volume of 1, 2, 3, 4, and 5 mL, an aliquot (100 μL) of CEST flour was added, followed by the addition of DPPH reagent (3 mL). The DPPH-extract mixture was then left at room temperature in the dark for 30 min, and change was observed based on 517 nm absorbance. Glutathione (GSH; 354102, Sigma-Aldrich) was used as a positive control. To ensure the validity of the data results, each sample was checked three times (triplicates test). Inhibition of DPPH was expressed as a percentage and determined according to the formula below:

$$\%\mathrm{DPPH}\mathrm{Inhibition}=\frac{\mathrm{A0}-\mathrm{A1}}{\mathrm{A0}}\times 100\%$$

A0 = Absorbance of blank; A1 = Absorbance of standard or sample.

### Combined eel and soy-based tempe experimental control trials study to evaluate hemoglobin levels, insulin-like growth factor I, and serum protein levels

**FIGURE 1:**
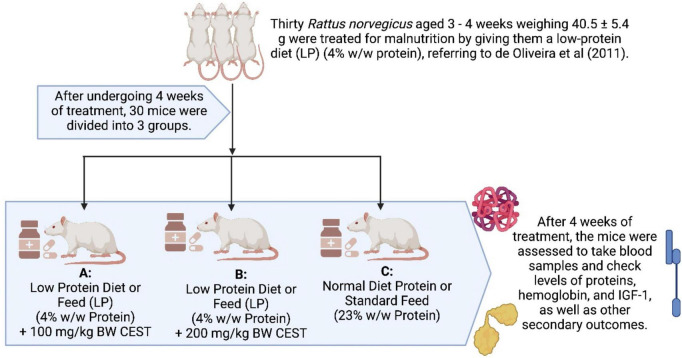
Study design (malnourished rats). The figure was using a legal and licensed BioRender.

A total of thirty (30) male white rats (*Rattus norvegicus*) aged 3–4 weeks weighing 40.5 ± 5.4 grams used in this study were given treatment in the form of malnutrition (malnutrition) conditions by giving a low-protein diet (LP) (4% w/w protein), referring to (21). After 4 weeks of treatment, the rats were divided into three groups. Group C (control) was given a regular diet of protein (NP) (23% w/w protein) *ad libitum* and without any dose of CEST. Rats in groups A and B stay on the same LP diet for 4 weeks but get a dose CEST of 100 and 200 mg/kg BW orally, respectively. This dose is based on the lower limit (low) and upper limit (high) for the safety of the rats’ stomachs. After 4 weeks of treatment, blood samples were analyzed for the levels of serum proteins, hemoglobin, and IGF-1. The flowchart of this study in detail is presented in Figure 1. The research protocol or use of experimental animals refers to the Declaration of Helsinki by the Council for International Organizations of Medical Sciences (CIOMS). In addition, this research protocol is performed following the Institutional Animal Care and Use Committee using the ARRIVE Guidelines and has been registered at Preclinical Trials Europe^1^ with the registration number of PCTE0000271 for providing ethical approval for the animal experiments in this research, and this study complies with all ethical regulations.

#### Rats’ blood sample collection

Throughout the experiment, all efforts were made to minimize the pain and suffering of experimental animals. After 4 weeks of treatment CEST Dosing, rats were on overnight fasting and given ketamine anesthesia to achieve this goal. A blood sample of 2.5 mL is collected from liver tissue and stored in a dry and clean tube (Tiger-Top tube) without the addition of anticoagulants to allow clotting at room temperature. Furthermore, the blood plasma sample was used to analyze the hemoglobin, hematocrit, and erythrocyte levels. The sample was then centrifuged for 20 min at a speed of 3,000 rpm which was collected to analyze protein, IGF-1, and Retinol-Binding Protein (RBP).

#### Biochemical analysis of rats’ blood samples

Hemoglobin (Hb) levels were tested using Rat Hemoglobin ELISA Kit (#ab157733) from a fresh blood plasma sample. Sample washed with phosphate-buffered saline (PBS, pH 7.4) 1% until the liquid becomes clear. Then, the sample is concentrated at a speed of 3,000 rpm for 20 min to get supernatant parts. The supernatant was taken to analyze the protein and IGF-I levels. Protein levels were measured using the Rat Protein Assay ELISA Kit (#MBS3808613). IGF-1 concentrations are estimated using the Rat IGF-1 ELISA Kit (#MBS268050) by the procedure manual at a wavelength of 450 nm. Rat erythrocyte protoporphyrin (EP) ELISA kit (RTES01121) was used to measure erythrocyte and hematocrit levels.

### Data management and statistical analysis

The data of amino acids and antioxidants was expressed by the standard deviation (SD, *n* = 3). A *p*-value of less than 0.05 (*p* < 0.05; 95% CI) is deemed statistically significant when using the ANOVA test to assess differences in data from various groups (for the analysis of primary outcomes and secondary outcomes). Paired or dependent *t*-test was conducted to determine the significant (*p* < 0.05; 95% CI) differences between the initial body weight (g) and Final Body Weight (g) of each group. An unpaired or independent *t*-test was conducted to determine the significant (*p* < 0.05; 95% CI) differences between GSH and WMGPY antioxidant scavenging activity. Using SPSS (Statistical Package for the Social Sciences) version 26 for MacBook, the data were statistically evaluated using homogeneity tests and MANOVA (Multivariate Analysis of Variance) testing. GraphPad Prism software version 9.2.0 was used to produce the graph visualizations. The illustration in the Graphical abstract uses a premium licensed Biorender belonging to one of the authors.

## Results

### Results of bioactive peptides and 2,2-diphenyl-1-picrylhydrazyl radical scavenging activity test

Six bioactive peptides (BPs) from CEST flour were successfully identified: Val-Glu-Glu (VEE, EBP1), Trp-Met-Phe-Asp-Trp (WMFDW, EBP2), Asp-Ala-Gly-Pro-Tyr-Gly-Pro-Ile (DAGPYGPI, BP3), Trp-Met-Gly-Pro-Tyr (WMGPY, BP4), Glu-Arg-Gly-Pro-Leu-Gly-Pro-His (ERGPLGPH, BP5) and Glu-Met-Gly-Pro-Ala (EMGPA, BP6) (shown in Table 1).

**TABLE 1:** Retention time (RT), molecular mass (Da), and amino acid sequences of six isolated bioactive peptides (BPs) (BP1 to BP6) from the CEST.

No.	RT (min)	Theoretical mass/observed mass (Da)	Amino acid sequence	Area (max)
BP1	9.05	375.37/375.38	VEE	188,602,459.32
BP2	11.36	783.89/783.91	WMFDW	128,180,581.48
BP3	12.63	788.84/788.83	DAGPYGPI	95,661,314.03
BP4	13.25	652.76/652.77	WMGPY	815,230,823.19
BP5	16.78	861.94/861.96	ERGPLGPH	312,358,366.23
BP6	17.22	503.57/503.59	EMGPA	204,192,224.93

RT, Retention times (minute); Theoretical Mass, the mass of amino acid sequence present in PubChem CID; Observed Mass, the mass of amino acid sequence obtained from the study; Area (max), peak abundance of amino acid sequence based on a mass spectrometer (MS).

**FIGURE 2:**
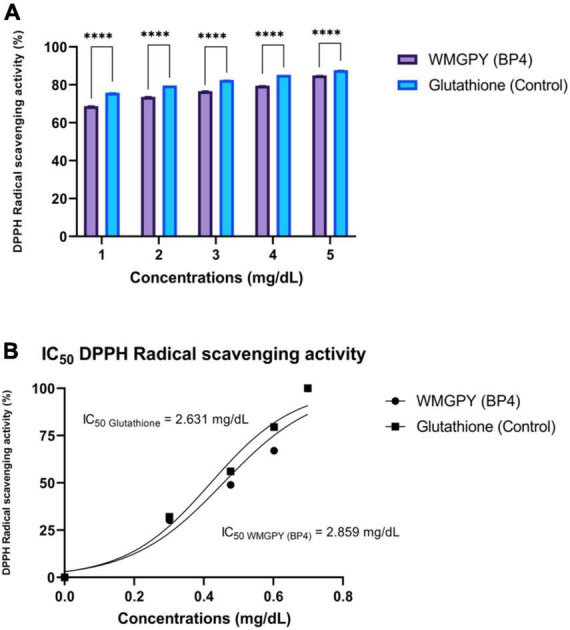
Antioxidant activity test. **(A)** Independent *t*-test results were conducted to determine the significant (*p* < 0.05; 95% CI) differences between GSH and WMGPY antioxidant scavenging activity, with ^*⁣*⁣**^ being a *p*-value less than 0.05 (Significant). **(B)** Dose-response curve of WMGPY and GSH regarding DPPH inhibition effect. IC_50_ = Half-maximal inhibitory concentration. The figure was using a legal and licensed BioRender.

Table 1 shows the obtained sequences of amino acids that act as bioactive peptides with their abundance. The most abundant BP in CEST flour is Trp-Met-Gly-Pro-Tyr (WMGPY, BP4), amounting to 815,230,823.19 (Table 1). Furthermore, the inhibitory activity of Trp-Met-Gly-Pro-Tyr (WMGPY, BP4) against DPPH was evaluated and compared with control or Glutathione (GSH). The results are shown in Figure 2. The chemical structure of the six BPs can be found in Supplementary file 1.

Figure 2 shows the results of an *in vitro* study inhibiting DPPH radical scavenging activity. The inhibitory activity of DPPH compared among the most abundant BP, namely WMGPY and GSH or glutathione. Results found that WMGPY showed lesser DPPH inhibition activities at doses 1, 2, 3, 4, and 5 mg/dL compared to GSH (*p* < 0.05). The DPPH inhibition of WMGPY was to close with GSH at a dose of 5 mg/dL with a percentage of 84.95 ± 0.06% and 87.73 ± 0.03%, respectively (Figure 2A). As shown in Figure 2B, WMGPY and GSH yield were IC_50_ of 2.85 and 3.63 mg/dL, respectively.

### Primary outcomes of an experimental control trials study

**FIGURE 3:**
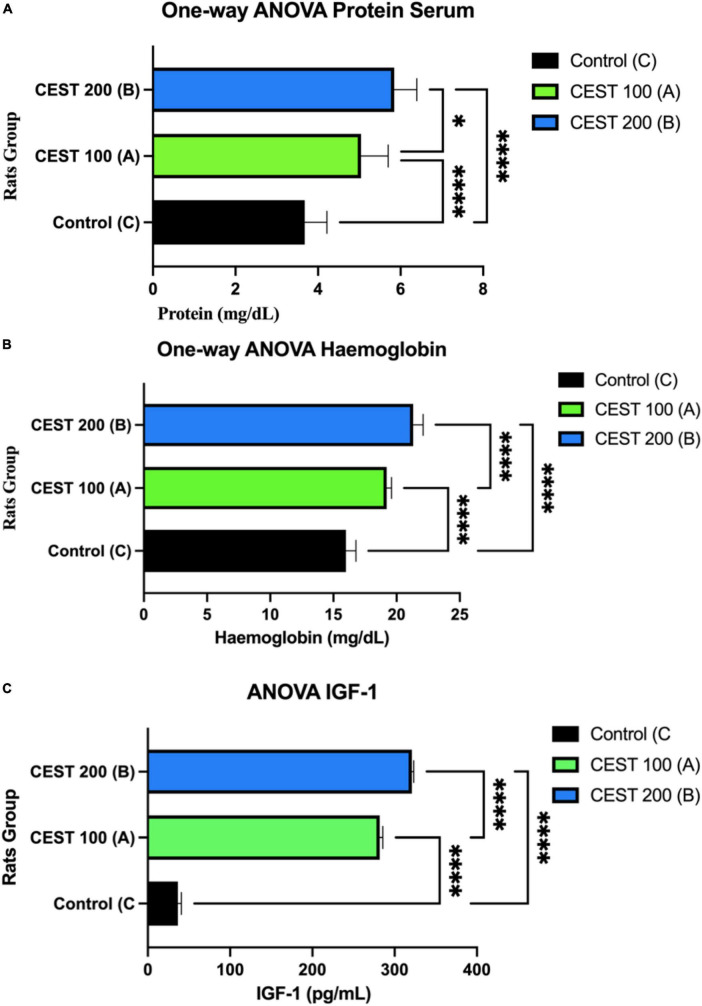
A higher dose of CEST significantly improves the level of serum protein, hemoglobin, and insulin-like growth factor I (IGF-1). **(A)** Comparison of serum proteins between treatment groups with * is the value *p* = 0.0128. **(B)** Comparison of hemoglobin between treatment groups. **(C)** Comparison of hemoglobin between groups of IGF-1 treatment, which indicates significance *p* < 0.0001 (One-way ANOVA Test; 95% CI). The figure was using a legal and licensed BioRender.

The results of the Levene test showed that the *p*-values for proteins, hemoglobin, and IGF-1 > 0.05, which means the same variance can be assumed as normal and homogeneous distributed data. Furthermore, the Multivariate ANOVA test showed a significant difference between the three variables of the three treatment groups (A, B, and C), *p* < 0.05 (Figure 3). The results also showed that serum protein was significantly lower in group C compared to groups A and B (*p* < 0.0001) (Figure 3A). Serum protein increased considerably in groups A and B (*p* = 0.0128) (Figure 3A). The effect of administering CEST at a dose of 200 mg/kg BW (Group B) is effectively higher than that of CEST at 100 mg/kg BW (Group A), to increase the serum protein of rats low-protein (LP) diet or malnourished, significantly (*p* < 0.05) (Figure 3A).

As expected, rats in group C or control (Normal Diet; NP) had significantly lower hemoglobin levels compared to groups A and B (*p* < 0.0001) (Figure 3B). In both groups A and B, hemoglobin increased significantly (*p* < 0.0001) in rats receiving a Low-Protein (LP) + CEST of 100 mg/kg BW (Group A), as well as Low-Protein (LP) + CEST 200 mg/kg BW treatment. Both doses of 100 and 200 mg/kg BW CEST increase hemoglobin, but doses of 200 mg/kg BW are more effective at increasing hemoglobin levels (*p* = 0.0001) (Figure 3B).

Group C had significantly lower serum IGF-1 levels or concentrations (Figure 3C). Serum IGF-1 concentrations were significantly higher in group B, as well as in group A than in group C. The effect of administration of CEST at a dose of 200 mg/kg BW (Group B) was more effective than CEST at 100 mg/kg BW (Group A), and the significant increase in serum IGF-1 in rats (*p* < 0.0001) (Figure 3A).

### Secondary outcomes of an experimental control trials study

The rat samples had similar characteristics between groups A, B, and C, with no significant differences in body weight before the intervention or initial body weight. Final body weight or after intervention turns out that there is no significant difference between groups. This means that LP is followed by a dose of 100 and 200 mg/kg of BW CEST equal to the body weight of rats on normal dietary protein (Group C). Furthermore, the levels of Erythrocytes and Hematocrit were significantly higher in groups A and B (the highest was in the B or Low-Protein (LP) + CEST 200 mg/kg BW treatment group) (Table 2). Similar to the previous result in this study, the highest RBP level was in group B, significantly (*p* < 0.0001). But, both doses of 100 and 200 mg/kg BW CEST increase RBP compared to group C or control (Table 2).

**TABLE 2:** Body weight characteristic, food, and water intake, feed efficiency ratio, erythrocytes and hematocrit of sample rats.

Groups	A (LP + 100 CEST)	B (LP + 200 CEST)	C (Normal/NP)	*P*-value**
Initial body weight (g)	44.72 ± 4.51	43.70 ± 3.44	42.37 ± 1.14	0.3041
Final body weight (g)	161.26 ± 7.14	162.51 ± 5.81	155.77 ± 4.49	0.0386
*P*-value*	<0.0001	<0.0001	<0.0001	
Weight gain (g/day)	4.16 ± 0.21	4.24 ± 0.21	4.05 ± 0.18	0.1131
Food intake (g)	6.30 ± 0.54	5.81 ± 0.64	5.65 ± 0.58	0.0514
Water intake (mL)	5.53 ± 0.49	5.92 ± 0.37	5.54 ± 0.56	0.1442
^a^FER (%)	66.44 ± 5.61	73.90 ± 9.28	72.52 ± 9.20	0.1156
Erythrocytes (×10^6^/μL)	6.38 ± 0.63	7.55 ± 0.95	5.47 ± 0.66	<0.0001
Hematocrit (%)	56.04 ± 4.57	61.19 ± 4.51	48.09 ± 2.46	<0.0001
^b^RBP (ng/mL)	6420.02 ± 168.96	7597.92 ± 211.52	5315.94 ± 122.00	<0.0001

*Paired t-test was conducted to determine the significant differences between each group’s initial body weight (g) and Final Body Weight (g).

**One-Way ANOVA was conducted to determine the significant differences of each parameter (Initial Body Weight (g), Final Body Weight (g), Weight Gain (g/day), Food Intake (g), Water Intake (mL), Food efficiency Ratio (FER, %), Erythrocytes and Hematocrit of each group.

^a^Food Efficiency Ratio (FER, %) = [Body weight gain (g/day)/food intake (g/day)] × 100, ^b^Retinol-Binding Protein (RBP, ng/mL).

## Discussion

### The potential of combined eel and soy-based tempe as a source of bioactive peptides and antioxidant activity

Proteins in foods sourced from agricultural aquaculture and their by-products have high structural diversity and are a considerable resource for exploring bioactive peptides (22). One food product that comes from aquaculture and has abundant protein and unsaturated fatty acids is Eel or Asian Swamp Eel (*Monopterus albus*) (1). Nevertheless, few studies have successfully explored and demonstrated the bioactive peptides component of *M. albus*. In addition to *M. albus*, agricultural products that are one of the superfoods in the world are tempe (fermented-soybeans by *Rhizopus* sp.) because of the high value of nutrients and the only plant-based food products containing B complex vitamins (23). Proteomic studies of bioactive peptide components from tempe have been done by Tamam et al. (24), and Val-His Peptides with Ala-Leu-Glu-Pro have been found in tempe from all manufacturers. Combined eel (*M. albus*) and soy-based tempe (CEST) in this combination can enrich bioactive peptides and their nutrients in the form of flour that can be used in various processed food products.

In previous basic studies, researchers have obtained CEST formulations with high levels of vitamin B9 and unsaturated fatty acids, followed by abundant amino acids and proteins. The nutritional facts profile was shown in Table 3 (3). Six bioactive peptides and their molecular weight and abundance have been identified from CEST (Tables 1, 3), with Trp-Met-Gly-Pro-Tyr (WMGPY, BP4) having a high level of abundance compared to the other 5 BPs. These six identified BPs have a role as antioxidant peptides in warding off free radicals (18). Previous literature suggests that the types of amino acids in BPs are considered a critical factor in their activity (25). Residual hydrophobic groups from hydrophobic amino acids such as Pro, Met, Ala, Leu, and Ile, can strongly react with hydrophobic PUFAs to inhibit lipid peroxidation in lipid-rich foods (26, 27). Wu et al. An inhibitory ability possessed by Pro-Met-Arg-Gly-Gly-Gly-Gly-Tyr-His-Tyr (PMRGGGGYHY) in free radical chain reactions is associated with Met residues because it can serve as a reactive site for formatting a sulfoxide structure for oxidation scavenge (28). The IC_50_ of WMGPY (BP4) from this study is higher than the research conducted by Zhang et al. (18) which only has an IC_50_ value of 0.38 mg/mL. Carboxyl and amino groups in polar amino acid residues are essential for capturing hydroxyl radicals and metal ion encroaching capacity of BPs (27, 29). In addition, Gly residues can maintain the high flexibility of the polypeptide skeleton, and its single hydrogen atom can be donated to neutralize Reactive Oxygen Species (ROS) (25, 30). Therefore, polar amino acids, including Asp residues in WMFDW, Gly residues in WMGPY, and Gly and Glu residues in EMGPA, have played an essential role in hydroxyl radical capture activities. CEST, which has an abundance of WMGPY followed by five other BPs, can be a source of free radical inhibition activity through the mechanism presented in the previous sentence.

**TABLE 3:** Nutrient composition, amino acids, bioactive peptides, and antioxidant scavenging activity of CEST.

Amino acids (mg/kg)	Proximate composition (%)	Calories (kcal/100 g)	Fatty acid (%)	Vitamin content (mcg/100 g)	Bioactive peptides	DPPH scavenging activity (mg/mL)
*L-Cysteine (6999.23 ± 4.91)	Protein content (57.08 ± 0.08)	From fat (225.41 ± 0.83)	Unsaturated (20.83 ± 0.12)	Vitamin B9 (1258.53 ± 1.39)	Val-Glu-Glu (VEE, BP1)	5.0 with IC_50_ = 2.85
*L-Methionine (2586.08 ± 0.71)	Ash content (2.61 ± 0.06)	Total (491.23 ± 0.12)	Saturated (4.22 ± 0.03)		Trp-Met-Phe-Asp-Trp (WMFDW, BP2)	
*L-Phenylalanine (31422.06 ± 156.91)	Total fat (25.05 ± 0.09)				Asp-Ala-Gly-Pro-Tyr-Gly-Pro-Ile (DAGPYGPI, BP3)	
*L-Isoleucine (23968.22 ± 52.86)	Moisture content (5.90 ± 0.02)				Trp-Met-Gly-Pro-Tyr (WMGPY, BP4)	
*L-Valine (24808.54 ± 177.79)	Carbohydrate (9.38 ± 0.26)				Glu-Arg-Gly-Pro-Leu-Gly-Pro-His (ERGPLGPH, BP5)	
*L-Arginine (39409.52 ± 201.39)					Glu-Met-Gly-Pro-Ala (EMGPA, BP6)	
*Glycine (29863.68 ± 136.47)						
*L-Lysine (31249.15 ± 179.35)						
*L-Leusin (41816.71 ± 169.84)						
*L-Tyrosine (20545.63 ± 91.08)						
*L-Proline (24809.83 ± 78.15)						
*L-Threonine (26498.19 ± 71.32)						
*L-Histidine (15231.22 ± 50.06)						
*L-Tryptophan (5314.78 ± 11.48)						
L-Serine (29646.04 ± 146.12)						
L-Glutamic (77122.75 ± 304.93)						
L-Alanine (24979.24 ± 106.30)						
L-Aspartic acid (46405.7 ± 175.86)						

*Essential and conditionally essential amino acid; without asterisk (*) non-essential amino acid.

### Nutritional status improving activity by combined eel and soy-based tempe supplementation

This preclinical or experimental control trial study evaluated the health benefits of processed food products in the form of CEST flour. This preclinical study is beneficial to find out the potential of CEST flour supplementation in increasing protein, hemoglobin, and IGF-1. This study is an *in vivo* or preclinical study that does not yet represent human results, but the dose produced in this study is beneficial as a reference for clinical trials in the subsequent study.

More than one-third of deaths of children under 5 years of age are related to malnutrition (31). Protein is one of the nutrients that play an essential role in repairing and building body tissues, such as allowing metabolic reactions to occur and coordinating body functions (32, 33). BPs that also act as amino acids were present in CEST and were strongly suspected of contributing to collagen formation for growth. The primary amino acids in collagen are glycine, alanine, proline, and glutamate acid (34). Collagen is an important component of connective tissue, which plays a vital role in the growth and healing process of wounds (35). Unsaturated fatty acid levels in CEST also play a role in increased protein metabolism-synthesis and muscle growth (as shown in Graphical Abstract). The increase in mTOR contributed by unsaturated fatty acids increases protein metabolism-synthesis and muscle growth (36).

The incidence of malnutrition, one of which is stunting, can be influenced by various factors due to the lack of macronutrients such as energy, protein, and fat (37). However, it is also influenced by the intake of micronutrients, minerals, and vitamins which also need to be considered (38). CEST has high vitamin B9 or folic acid (Table 3). Vitamin B9 plays an essential role in purine and thymidylate syntheses in forming red blood cells or effective erythropoiesis (Graphical abstract) (39). This was in line with the results of this study that administering CEST doses of 200 mg/kg BW (Group B) can increase hemoglobin, erythrocyte, hematocrit, and RBP levels in malnourished rats, significantly (Figure 3B and Table 2). Furthermore, RBP is the main carrier of vitamin A in blood (40). Children with vitamin A deficiency will experience growth disorders (stunting) (40). CEST improved RBP levels in malnourished rats, which may potentially prevent stunting in children. The increase in RBP levels was also observed in humans supplemented with high-protein food (41). In addition, BPs-antioxidants in CEST were also thought to have hemoglobin’s protective properties from free radical damage (Graphical Abstract).

In line with increased protein and hemoglobin results, IGF-1 was significantly higher in malnourished rats given CEST doses of 200 mg/kg BW (Group B) (Figure 3C). This result was in line with a systematic review and meta-analysis that increased protein intake was significantly associated with an increase in circulating IGF-1 levels in humans (42). Children and adolescents of short stature usually suffer from low levels of Growth Hormone (GH) (43, 44). One such GH is insulin-like growth factor-1 (IGF-1), which plays a vital role during critical periods of a child’s growth and development (45, 46). Previous research has shown that GH/IGF-1 disorder was more common in children of short stature (47). The increase in IGF-1 levels in rats given CEST was thought to be due to the presence of BPs (related to antioxidant activity) and levels of nutrients and vitamins that were already present in CEST (Table 3), such as B9 or folic acid. Supported by other studies, antioxidant effects can contribute to maintaining blood cell-vessel integrity by counteracting oxidative stress, thereby limiting the development of cholesterols accumulation-induced atherosclerosis and increasing IGF-1 (40, 48).

However, it is thought that there is a relation between CEST (as a combination of fermented products with synbiotic properties, mainly from tempe) and its benefits or effect on the gut microbiome, which is also thought to contribute to improved nutritional status (Graphical Abstract). This research still needs to be developed again to support the claim that CEST can be a functional food for malnourished children and adolescents. Furthermore, there’s a possibility of bioactive peptides which were discovered through the enzymatic hydrolysis method, which is predicted to bring richer results and will be considered for further study. CEST has the potential to become a functional food that can improve nutritional status, and this dose can be used as a reference for clinical trials. Therefore, further studies need to be done to examine its effects on the gut microbiome.

## Conclusion

Six bioactive peptides with Trp-Met-Gly-Pro-Tyr (WMGPY, BP4) are most abundant in a combined eel and tempe (CEST) flour. They have been successfully identified, and their association improves nutritional status biomarkers by ameliorating serum proteins, hemoglobin, and IGF-1. Based on these experimental control trials, 200 mg/kg of BW is the recommended dose for follow-up clinical trial research. This implies that the CEST flour has six bioactive peptides whose potential as functional food may improve the nutritional status biomarkers explored (as shown in Graphical Abstract).

## Data availability statement

The raw data supporting the conclusions of this article will be made available by the authors, without undue reservation.

## Ethics statement

This animal study was reviewed and approved by the Pre-clinical Trials Europe (www.preclinicaltrials.eu). The research protocol or use of experimental animals refers to the Declaration of Helsinki by the Council for International Organizations of Medical Sciences (CIOMS). In addition, this research protocol is performed following the Institutional Animal Care and Use Committee using the ARRIVE Guidelines and has been registered at Pre-clinical Trials Europe (www.preclinicaltrials.eu) with the registration number of PCTE0000271 for provided ethical approval for the animal experiments in this research, and this study complies with all ethical regulations.

## Author contributions

NS, FN, and HP conducted the experiments, analyzed the data, wrote the manuscript, designed the research, and conceptualized the ideas. MR, NT, HH, WG, MH, NM, SR, and DP contributed to the data analysis, critiquing manuscripts, interpreting manuscript results, and editing. MT, SN, and VY assisted in the processing of data and helped to revise and graphical abstract editing. All authors contributed to the article and approved the submitted version.

## Footnotes

1
www.preclinicaltrials.eu

## Abbreviations

Ala: Alanine
Arg: Arginine
Asp: Aspartic acid
BPs: Bioactive Peptides
BW: Body Weight
CEST: Combined eel (*M. albus*) and soy-based tempe
CIOMS: Council for International Organizations of Medical Sciences
DAGPYGPI: Asp-Ala-Gly-Pro-Tyr-Gly-Pro-Ile
DPPH: 2,2-diphenyl-1-picrylhydrazyl
EMGPA: Glu-Met-Gly-Pro-Ala
ERGPLGPH: Glu-Arg-Gly-Pro-Leu-Gly-Pro-His
ESI: Electrospray ionization
Glu: L-glutamate or Glutamic acid
Gly: Glycine
GSH: Glutathione
His: Histidine
IGF-1: insulin-like growth factor-1
Ile: Isoleucine
Leu: Leucine
LP: Low-protein
Met: Methionine
MS: mass spectrometer
mTOR: Mechanistic target of rapamycin
NP: Normal-protein
Phe: Phenylalanine
Pro: Proline
PUFAs: Polyunsaturated fatty acids
RBP: Retinol-Binding Protein
ROS: Reactive Oxygen Species
Trp: Tryptophan
Tyr: Tyrosine
Val: Valine
VEE: Val-Glu-Glu
WMFDW: Trp-Met-Phe-Asp-Trp
WMGPY: Trp-Met-Gly-Pro-Tyr.
