# Supplementary material for: Bioactive peptides identification and nutritional status ameliorating properties on malnourished rats of combined eel and soy-based tempe flour
Source: Front Nutr. 2022 Sep 28;9:963065. doi: 10.3389/fnut.2022.963065 (PMC9554436; doi:10.3389/fnut.2022.963065)
Supplement: Supplementary file 1 [file Table_1.DOCX]

Supplementary Material

Supplementary 1. The chemical structure of the six BPs

| No. | Chemical Structure | Amino Acid Sequence |
| --- | --- | --- |
| BP1 |  | VEE |
| BP2 |  | WMFDW |
| BP3 |  | DAGPYGPI |
| BP4 |  | WMGPY |
| BP5 |  | ERGPLGPH |
| BP6 |  | EMGPA |
